# The Beneficial Impact of Intraoperative Ultrasound on Resection Margin Status during Breast Conserving Surgery

**DOI:** 10.1155/2022/2268821

**Published:** 2022-12-07

**Authors:** Osama Almezaien, Ahmed Mohamed Eldeeb, Abdelfattah Kalmoush, Mohamed Shaaban Nassar, Tarek Zaghlol Mohamed, Mohamed Sobhy Shaaban, Mohamed Ibrahim Henish, Sobhy Teama, Saed Abdolmonem Elgohary Khalafallah, Lofty A. Ibrahim

**Affiliations:** ^1^Department of General Surgery, Faculty of Medicine, Al-Azhar University, Cairo, Egypt; ^2^Department of Radiology, Faculty of Medicine, Al-Azhar University, Cairo, Egypt

## Abstract

**Background:**

Surgical resection with clear surgical cut margins is the mainstay of managing malignant breast neoplasms. Multiple techniques have been suggested to enhance resection status during breast-conserving surgery (BCS), including intraoperative ultrasonography (IOUS). Herein, we conducted the current investigation to reveal the benefit of IOUS on the achievement of R0 resection. *Patients and Methods*. This retrospective investigation included 140 patients who underwent BCS. They were divided into two groups: the IOUS group (40 cases) and the control group (100 cases). Our primary objective was to determine the free resection margin status (R0).

**Results:**

Both study groups expressed statistically comparable demographic and clinical data. Additionally, histopathological examination revealed no significant difference between the two groups regarding the tumor type, stage, or grade. Nonetheless, the R0 resection margin was more frequently encountered in association with IOUS application (97.5% compared to 79% in the control group), and that difference was statistically significant (*p*=0.007).

**Conclusion:**

The application of IOUS has a significant beneficial impact on the outcomes of BCS. It is associated with a marked decline in positive resection margins, and its application should be encouraged in the breast oncological practice.

## 1. Introduction

In Egypt, breast cancer is the most common malignant neoplasm detected in the female population, accounting for about 30% of malignant neoplasms in this population [1]. Thanks to effective screening programs and increased public awareness, breast cancer patients are often detected at early stages [2, 3].

Surgical resection is still the main management method for breast cancer, and breast-conserving surgery (BCS) is preferable for patients who have earlier stages [4]. The primary goal of surgery is to obtain cut margins free of tumor tissue. Infiltrated margins are associated with high postoperative recurrence rates, which will need re-excision, mastectomy, or even adjuvant radiotherapy [5–7]. Therefore, it is crucial to achieve clear surgical cut margins during BCS to enhance patients' oncological outcomes [8].

Multiple methods have been applied for better tumor localization during BCS, like radioactive seeds, wire placement, and intraoperative ultrasonography (IOUS). The former two methods have their own drawbacks. The implementation of radioactive seeds needs to be one day before surgery. It is also an invasive procedure that has a high financial cost [9]. Likewise, wire localization needs to be done in a separate setting before surgery, and it is associated with increased patient anxiety [10].

Contrarily, IOUS is widely available in most surgical theatres nowadays. It could be done in the same operative setting without scheduling problems or increased exposure to radiation [11–13]. IOUS of the excised surgical specimen could be used to ensure appropriate margins surrounding the whole specimen. If a deficient margin is detected the surgeon is informed and a re-excision of the infiltrated region is performed within the same primary setting [9, 14].

The previous technique has recently been adopted in our center, especially with the lack of other expensive localization techniques in a resource-limited country like Egypt. Thus, the current investigation was conducted to reveal the benefit of IOUS on the achievement of R0 resection.

### 1.1. Patients and Methods

This retrospective investigation was carried out at the Al-Azhar University General Surgery Department following approval from the local scientific committee of the same university. We collected the data of all female patients who underwent BCS for breast cancer at our institute during the period between January 2019 and December 2021. Women who had bilateral lesions or who underwent wire-guided surgery or mastectomy were excluded.

All patients were subjected to history taking (including risk factors for cancer, family history, and previous neoadjuvant therapy), a local breast examination, and a routine preoperative laboratory workup. Other investigations included breast mammography, US, and US-guided core biopsy.

The allocated 140 patients were divided into two groups according to the use of IOUS: the IOUS group included 40 patients, and the control group included the remaining 100 cases. The BCS was performed by an experienced surgeon under general anesthesia, and these procedures included quadrantectomy, segmental resection, or lumpectomy. The choice of the operation was mainly dependent on the operator's choice. The excised specimen was marked by silk sutures to be correctly oriented for the subsequent radiological or histopathological examinations.

In the IOUS group, the specimen was immersed in a water bath and then scanned by the US probe of the Toshiba Aplio 500 device using the linear probe (10 MHz) by a radiologist experienced in breast ultrasonography. Images were obtained for the six surfaces of the surgical specimen, and the surgeon was informed if the tumor tissue infiltrated the cut margin or if the resected margin was too small or unclear. These cases were managed by re-excision in the same setting in the same direction of the infiltrated or suspected surface.

After surgery, the specimen was sent for histopathological analysis. Tumor type, grade, and stage were assessed. In addition, the surgical cut margin was classified as negative when the distance between the tumor and the margin was ≥1 mm (R0) or positive when it was infiltrated by tumor tissue (R1) [15]. The histopathological findings were considered the standard gold measurement of cut margin infiltration in the current study.

The primary outcome of our investigation was to elucidate if the application of IOUS helped in the increase in R0 detection rates or not.

### 1.2. Statistical Analysis

The Statistical Package for Social Science (version 27 for Windows® (IBM SPSS Inc., Chicago, IL, USA) was utilized for data tabulation and analysis. Quantitative data were tested for normality using the Shapiro–Walk test and then expressed as the mean ± SD or median (min-max). To compare two independent groups of parametric quantitative data, independent samples *t*-test was used, and compares two independent groups of nonparametric quantitative data, independent samples *t*-test Qualitative data were expressed as numbers and relative percentages. To compare two independent groups of qualitative data, the chi-square (Fischer's exact test or Monte-Carlo test) were used as appropriate. A *p* value of 0.05 or less was considered to be significant.

## 2. Results

The included women had mean ages of 46.5 and 45.26 years in the IOUS and control groups, respectively. Diabetes mellitus was present in 7.5% and 6% of patients, while hypertension was reported by 7.5% and 5% of patients in the same two groups, respectively. Only one patient (1%) had chronic kidney disease in the control group. Previous chemotherapy was received in 22.5% and 26% of cases, whereas hormonal therapy was commenced for 5% and 8% of patients in the same two study groups, respectively. All of the previous variables were statistically comparable between the two study groups (*p* > 0.05) Table 1.

Histopathological analysis of the excised specimen revealed invasive ductal adenocarcinoma in most of the study cases (87.5% and 81% of patients in the two study groups, respectively), followed by invasive lobular carcinoma (10% and 12% of patients in the same two groups, respectively). Other findings included tubular and medullary carcinomas.  Table 2 shows the absence of any significant differences between the two groups regarding the previous parameter.

According to the pathological TNM classification (pTNM), most patients had the T1 stage (87.5% and 81% in the two groups, respectively), while the T2 stage was present in 10% and 16% of patients in the same two groups, respectively. The remaining cases had the T3 stage (Table 3).

Tumor grade was also comparable between the two study groups (Table 4). Grade 2 was the most common one (70% and 76% of patients in the two groups, respectively), followed by grade 1 (22.5% and 14% in the same groups, respectively). The remaining cases had grade 3 lesions.

When it comes to our primary outcome, the application of IOUS was associated with higher R0 resection rates (97.5% versus 79% in controls—*p* = 0.007) (Table 5). IOUS application led to a change in the resection limits in four patients (10%), as US assessment revealed infiltrated margin in these patients, and they were managed by re-excision in the same primary setting.

## 3. Discussion

Complete excision of breast cancer is still the main step in the management of such pathology, in spite of great advances in the methods of diagnosis and treatment. BCS is preferred by surgeons for localized lesions as it is associated with better cosmetic outcomes and patient satisfaction. Also, it has a comparable oncological outcome to the radical mastectomy operation when performed in selected patients. However, some malignant breast neoplasms have microscopic spread that may not be noticed by the surgeon intraoperatively and detected on the subsequent pathological analysis of the specimen [14, 16].

These patients require additional interventions for the residual tumor tissue, including re-excision. Therefore, it is crucial to minimize this scenario to improve oncological outcomes, decrease the need for reoperation, and decrease financial healthcare costs [17, 18].

The application of IOUS was greatly beneficial for this dilemma, and that was proved in the current investigation. First of all, one could notice the absence of any significant differences between the IOUS and control groups regarding most of the tumor criteria, despite the retrospective nature of our study. That should reduce any bias skewing our results in favor of the IOUS group rather than the controls. Our findings revealed a significant increase in R0 resection margins in association with IOUS application (97.5% vs. 79% in controls—*p* < 0.05). These findings should have a positive impact on postoperative oncological outcomes and the need for reoperation, although not being studied in our investigation.

Eichler and his associates agreed with our findings, as R0 resection was obtained in 96.4% of IOUS patients, compared to 82.5% of controls, with a significant difference between them (*p* < 0.05) [14]. Moore et al. also reported that only one patient (3.5%) in the IOUS group had infiltrated margins, compared to seven patients (29%) in the control group, with a significant difference in statistical analysis (*p* < 0.05) [19].

Krekel et al. also noticed a significant decline in resection margin involvement with IOUS application (*p* = 0.009). Infiltrated margins were encountered in 3% and 17% of patients in the IOUS and control groups, respectively [20].

Vispute et al. reported that positive surgical cut margins were more encountered in the control group (14.28%) compared to only 3.22% when the IOUS was used. Despite that difference, the statistical analysis revealed its nonsignificance [16]. Moreover, Oshla and his colleagues reported that IOUS was helpful in decreasing reoperation rates by localization of neoplastic tissue in the resected area and thus, achieving R0 margins [21].

In our study, although one patient (2.5%) was missed by IOUS and proved to have infiltrated margins on the pathological examination, another four patients (10%) were discovered by the same modality to have infiltrated margins, and they were managed by re-excision in the same operative setting. Although the failure rate was too low (2.5%), it is expected to improve with the increased learning curve with more US specimen examinations.

The current study has some limitations; being retrospective in nature, along with its application in a single medical center, are the main drawbacks. This should encourage researchers to conduct more studies, including more patients from different oncological centers in the near future.

## 4. Conclusions

The application of IOUS has a significant beneficial impact on the outcomes of BCS. It is associated with a marked decline in positive resection margins, and its application should be encouraged in the breast oncological practice.

## Figures and Tables

**Table 1:** Patient demographic and clinical criteria in the two study groups.

Variables	IOUS groups (*n* = 40)	Control groups (*n* = 100)	*p* values
Age (years)	46.50 ± 7.73	45.26 ± 8.46	0.424

*Comorbidities*			
(i) Diabetes	3 (7.5%)	6 (6%)	0.744
(ii) Hypertension	3 (7.5%)	5 (5%)	0.565
(iii) Chronic kidney disease	0 (0%)	1 (1%)	0.526

*Previous treatment*			
(i) Chemotherapy	9 (22.5%)	26 (26%)	0.666
(ii) Hormonal therapy	2 (5%)	8 (8%)	0.534

**Table 2:** Tumor histopathology in the two study groups.

Histopathology	IOUS groups (*n* = 40)	Control groups (*n* = 100)	*p* values
(i) Invasive ductal carcinoma	33 (82.5%)	86 (86%)	
(ii) Invasive lobular carcinoma	4 (10%)	12 (12%)	
(iii) Tubular carcinoma	2 (5%)	2 (2%)	0.315
(iv) Medullary carcinoma	1 (2.5%)	0 (0%)	

**Table 3:** Tumor pathological stage in the two study groups.

pTNM stages	IOUS groups (*n* = 40)	Control groups (*n* = 100)	*p* values
(i) T1	35 (87.5%)	81 (81%)	
(ii) T2	4 (10%)	16 (16%)	0.641
(iii) T3	1 (2.5%)	3 (3%)	

**Table 4:** Tumor grade in the two study groups.

pTNM grades	IOUS groups (*n* = 40)	Control groups (*n* = 100)	*p* values
(i) Grade 1	9 (22.5%)	14 (14%)	
(ii) Grade 2	28 (70%)	76 (76%)	0.452
(iii) Grade 3	3 (7.5%)	10 (10%)	

**Table 5:** Resection margin according to the final histopathological examination.

Resection status	IOUS groups (*n* = 40)	Control groups (*n* = 100)	*p* values
(i) R0	39 (97.5%)	79 (79%)	0.007^*∗*^
(ii) R1	1 (2.5%)	11 (11%)	

## Data Availability

The data used to support the findings of this study are available from corresponding authors upon request.

## References

[1] Ibrahim A. S., Khaled H. M., Mikhail N. N., Baraka H., Kamel H. (2014). Cancer incidence in Egypt: results of the national population-based cancer registry program. *J Cancer Epidemiol*.

[2] Coleman C. (2017). Early detection and screening for breast cancer. *Seminars in Oncology Nursing*.

[3] da Costa Vieira R. A., Biller G., Uemura G., Ruiz C. A., Curado M. P. (2017). Breast cancer screening in developing countries. *Clinics*.

[4] Sun Z. H., Chen C., Kuang X. W., Song J. L., Sun S. R., Wang W. X. (2021). Breast surgery for young women with early-stage breast cancer: mastectomy or breast-conserving therapy?. *Medicine (Baltimore)*.

[5] Aziz D., Rawlinson E., Narod S. A. (2006). The role of reexcision for positive margins in optimizing local disease control after breast-conserving surgery for cancer. *Breast Journal*.

[6] Cellini C., Huston T. L., Martins D. (2005). Multiple re-excisions versus mastectomy in patients with persistent residual disease following breast conservation surgery. *The American Journal of Surgery*.

[7] Luini A., Rososchansky J., Gatti G. (2009). The surgical margin status after breast-conserving surgery: discussion of an open issue. *Breast Cancer Research and Treatment*.

[8] Lee J., Park H. Y., Kim W. W. (2020). Clinical efficacy of intraoperative ultrasound for margin assessment in breast-conserving surgery. *Breast Journal*.

[9] Chakedis J. M., Tang A., Kuehner G. E. (2021). Implementation of intraoperative ultrasound localization for breast-conserving surgery in a large, integrated health care system is feasible and effective. *Annals of Surgical Oncology*.

[10] Hayes M. K. (2017). Update on preoperative breast localization. *Radiologic Clinics of North America*.

[11] Volders J. H., Haloua M. H., Krekel N. M. (2017). Intraoperative ultrasound guidance in breast-conserving surgery shows superiority in oncological outcome, long-term cosmetic and patient-reported outcomes: final outcomes of a randomized controlled trial (COBALT). *European Journal of Surgical Oncology*.

[12] Krekel N. M., Lopes Cardozo A. M., Muller S., Bergers E., Meijer S., van den Tol M. P. (2011). Optimising surgical accuracy in palpable breast cancer with intra-operative breast ultrasound--feasibility and surgeons’ learning curve. *European Journal of Surgical Oncology*.

[13] Pan H., Wu N., Ding H. (2013). Intraoperative ultrasound guidance is associated with clear lumpectomy margins for breast cancer: a systematic review and meta-analysis. *PLoS One*.

[14] Eichler C., Hübbel A., Zarghooni V. (2012). Intraoperative ultrasound: improved resection rates in breast-conserving surgery. *Anticancer Research*.

[15] Eggemann H., Ignatov T., Costa S. D., Ignatov A. (2014). Accuracy of ultrasound-guided breast-conserving surgery in the determination of adequate surgical margins. *Breast Cancer Research and Treatment*.

[16] Vispute T., Seenu V., Parshad R. (2018). Comparison of resection margins and cosmetic outcome following intraoperative ultrasound-guided excision versus conventional palpation-guided breast conservation surgery in breast cancer: a randomized controlled trial. *Indian Journal of Cancer*.

[17] Barellini L., Marcasciano M., Lo Torto F., Fausto A., Ribuffo D., Casella D. (2020). Intraoperative ultrasound and oncoplastic combined approach: an additional tool for the oncoplastic surgeon to obtain tumor-free margins in breast conservative surgery-A 2-year single-center prospective study. *Clinical Breast Cancer*.

[18] Pop M. M., Cristian S., Hanko-Bauer O., Ghiga D. V., Georgescu R. (2018). Obtaining adequate surgical margin status in breast-conservation therapy: intraoperative ultrasound-guided resection versus specimen mammography. *Medicine and Pharmacy Reports*.

[19] Moore M. M., Whitney L. A., Cerilli L. (2001). Intraoperative ultrasound is associated with clear lumpectomy margins for palpable infiltrating ductal breast cancer. *Annals of Surgery*.

[20] Krekel N. M., Haloua M. H., Lopes Cardozo A. M. (2013). Intraoperative ultrasound guidance for palpable breast cancer excision (COBALT trial): a multicentre, randomised controlled trial. *The Lancet Oncology*.

[21] Olsha O., Shemesh D., Carmon M. (2011). Resection margins in ultrasound-guided breast-conserving surgery. *Annals of Surgical Oncology*.

